# Transport Phenomena in Gel

**DOI:** 10.3390/gels2020017

**Published:** 2016-05-11

**Authors:** Masayuki Tokita

**Affiliations:** Department of Physics, Faculty of Science, Kyushu University, 744 Motooka, Fukuoka, Fukuoka 812-8581, Japan; tokita@phys.kyushu-u.ac.jp; Tel.: +81-92-802-4095

**Keywords:** diffusion, friction, scaling theory, colloid gel

## Abstract

Gel becomes an important class of soft materials since it can be seen in a wide variety of the chemical and the biological systems. The unique properties of gel arise from the structure, namely, the three-dimensional polymer network that is swollen by a huge amount of solvent. Despite the small volume fraction of the polymer network, which is usually only a few percent or less, gel shows the typical properties that belong to solids such as the elasticity. Gel is, therefore, regarded as a dilute solid because its elasticity is much smaller than that of typical solids. Because of the diluted structure, small molecules can pass along the open space of the polymer network. In addition to the viscous resistance of gel fluid, however, the substance experiences resistance due to the polymer network of gel during the transport process. It is, therefore, of importance to study the diffusion of the small molecules in gel as well as the flow of gel fluid itself through the polymer network of gel. It may be natural to assume that the effects of the resistance due to the polymer network of gel depends strongly on the network structure. Therefore, detailed study on the transport processes in and through gel may open a new insight into the relationship between the structure and the transport properties of gel. The two typical transport processes in and through gel, that is, the diffusion of small molecules due to the thermal fluctuations and the flow of gel fluid that is caused by the mechanical pressure gradient will be reviewed.

## 1. Introduction

Gel has the characteristics and common structure that consists of the three-dimensional polymer network and gel fluid. Because of such a diluted structure, many molecules are transported through gel. Such a characteristic of gel is used in separation technologies, namely, gel electrophoresis, gel permeation chromatography, and so forth. Therefore, information on the transport phenomena in and across gel is of importance in designing a separation system. Moreover, knowledge on transport phenomena in gel has become quite important recently for understanding the volume phase transition of gel and the pattern formation phenomena of gel [1,2,3]. It has been reported that the volume of gel reversibly changes more than a thousandfold in response to the slight environmental change in the vicinity of the volume phase transition point [4]. The huge amount of gel fluid, therefore, flows in and out in accordance with the swelling and shrinking of gel. Kinetic study on the volume change of gel indicates that the characteristic time of the swelling of gel, *τ*, is determined by the ratio of the longitudinal modulus of gel, *E*, the friction between gel fluid and the polymer network of gel, *f*, and the typical size of gel, *a*, as $\tau =a^{2}f/E$ [5]. In contrast, the shrining pattern formation phenomena in the shrinking process of gel in the poor solvent is very complicated since the first event that occurs in the beginning of the shrinking process is the diffusion of the poor solvent into gel. Then, the squeeze out of gel fluid came in to create the shrinking patterns. Both the diffusion and the friction compete with each other in pattern formation in the shrinking gels. As a result, various shrinking patterns appear in the shrinking gel [6,7,8]. In addition to this, one of the most striking expectations is deduced from the study of the critical dynamics of the volume phase transition of gel, namely, the friction of gel becomes smaller and it vanishes at the volume phase transition point [9]. The diffusion and the friction, therefore, play important roles in the volume phase transition of gel as well as the pattern formation in gel. The detailed studies on the transport of substances by the diffusion and the flow of gel fluid are still required for the full understanding of the volume phase transition of gel and the pattern formation in gel. Since the polymer network of gel is regarded as an assembly of the molecular mesh, the relationship between the transport properties of gel and the mesh size of the polymer network of gel should first be clarified. The diffusion of small molecules in gel and the frictional properties of gel have been studied by many researchers so far [10,11,12,13]. Although many studies have been conducted, most measurements are made under not ideal conditions. In this review, we will discuss the diffusion and the friction of gel in terms of the structure of gel by which the simple view for the transport phenomena in gel will be deduced.

## 2. Gel

Gel is classified into two classes by the structure of the cross-linking point. One is called chemical gel and the other is known as physical gel. In the case of chemical gel, the cross-linking point consists of a covalent bond. On the other hand, the cross-linking point of physical gel consists of the assembly of polymers that is formed by the short range interactions such as the van der Waals interaction, the hydrophobic interaction, the hydrogen bond, and the Coulomb interaction between charges. The network structure of chemical gel is not varied by the change of the external environment such as the temperature, because the bonding energy of the covalent bond is much higher than the thermal energy. Hence, the cross-linking point of the polymer network is unchanged even if the temperature is changed, say, from 0 to 100 ${}^{\circ }$C. Gel is in an equilibrium swelling state [14]. The volume of gel changes in response to environmental changes such as the temperature in the case of chemical gel. If the appropriate chemical structure is given to the polymer network of gel, then gel shows the volume phase transition phenomena. On the other hand, the bonding energy due to short range interactions is almost the same order of magnitude as the thermal energy. Accordingly, most physical gels show the sol-gel transition phenomena when the environmental conditions are changed. The sol-gel transition and the volume phase transition are the characteristic transitions in gel system [15].

In this review, we describe the transport properties of the chemical gels of acrylamide (AAm) and *N*- (NIPA) cross-linked by *N,N*-methylenebisacrylamide (BIS). The linear polymer chains of AAm or NIPA are connected by BIS to form a three-dimensional polymer network. The network structure is, therefore, determined both by the concentrations of the linear chain component and the cross-linker. The cross-linking density of gel, which is defined by the mole fraction of the cross-linker to the total amount of the linear component and the cross-linker, is fixed at 0.01 or 0.02 in most studies. The total concentration of gel is changed under the fixed cross-linking density. The gels obtained under these conditions are uniform; hence, gel is transparent and elastic. On the other hand, the cross-linking density is changed at a constant total concentration, usually fixed at 700 mM. The gel prepared at higher concentrations of BIS are opaque and brittle suggesting the formation of a non-uniform structure.

The mesh size of the polymer network is the most important parameter that defines the structure of the polymer network of gel. The gels used here are synthesized by the random copolymerization of the linear chain component and the cross-linker. As a result of this, the network structure of gel becomes more or less random. The size of the polymer mesh is, therefore, expressed by an averaged parameter that is called the correlation length of gel, *ξ*. The correlation length of gel represents, in a good solvent, the distance between two nearest neighbor contact points of polymers. The correlation length of gel varies with the concentration of the linear chain component, the cross-linking density, and the environmental conditions such as the temperature.

For most parts of the study, poly(acrylamide) gel (PAAm gel) is chosen as the sample gel since this gel is widely used in separation and the purification technologies. Hence, the physical and the chemical properties of PAAm gel are well known. For instance, the concentration of gel can be changed widely from 0.02 to 0.5 g/mL because of a high affinity with water. This is a definite advantage for the present purpose of the study because the correlation length of the polymer network of gel can be changed widely. In addition to this, PAAm gel does not interact strongly with many water soluble molecules. This is another great advantage for the present study since it widens the choice of the probe molecules. On the other hand, poly(n-siopropylacrylamide) gel (PNIPA gel) is known as the thermo-sensitive gel. The gel shows the discontinuous volume change at about 34 ${}^{\circ }$C in water. The correlation length of PNIPA gel can be changed reversibly as a function of the temperature. Therefore, the critical behaviors of the frictional property can be revealed by studying the frictional property of PNIPA gel in the vicinity of the volume phase transition point.

## 3. Probe Diffusion in Gel

Many advancements in nuclear magnetic resonance technology have been made at the end of the last century. One of the useful technologies in the study of transport phenomena is the pulsed field gradient nuclear magnetic resonance method (PFG-NMR). The diffusion coefficient of the specific molecules, which will be called the probe molecule hereafter, can be determined by using PFG-NMR invasively if the probe molecule is distinguishable in the NMR spectrum of the whole system. The PGF-NMR method is firstly applied to study the diffusion of the molecules in simple solution. It is then applied to complex systems such as the semi-dilute polymer solution systems, and finally to polymer gels [10,16,17]. Previous studies suggest that it is a requirement to study the diffusion process by changing both the concentration of gel and the size of the probe molecule systematically to obtain the entire aspects of the diffusion process in gel. The diffusion of the probe molecule in gel is, however, extensively affected by short range interactions between the probe molecule and the polymer network. The presence of short range interactions is unfavorable because we firstly focus our attention only on the effects of the polymer network on the diffusion process of the probe molecules. Since the short range interactions depend strongly on the chemical structures of the polymer network and the probe molecules, one can minimize the complex situations by choosing appropriate chemicals for gel and the probe molecules. Then, we can deduce the effects of only the polymer network of gel on the diffusion of the probe molecules.

In this study, the diffusion coefficient of the probe molecules are measured in PAAm gel of the total concentration from 0.02 to 0.5 g/mL at a cross-linking density of 0.02. Although any substance can be a candidate for the probe diffusion experiments in PAAm gel, there are still several requirements for the probe molecule for the present purpose of the study as listed below.

1. Highly soluble in water because water is used as a solvent.
2. Compact molecules to be assumed as a spherical molecule.
3. Molecular weight spreads at least one order of magnitude.
4. Chemical shift does not overlap with that of PAAm gel.
5. Absence of strong interaction with the polymer network of PAAm gel.

Taking into account these requirements, we finally choose water (solvent), ethanol, glycerin, poly(ethylene glycol), and sucrose as the probe molecules. The molecular weight of each probe molecule is, 18, 46, 92, 200, and 342, respectively.

The sample PAAm gel is synthesized in an NMR tube of 10 mm in diameter. The solvent is the mixture of water and the heavy water ($H_{2}O$:$D_{2}O$ = 9:1) which contains the probe molecule at a concentration of 10 wt % is prepared firstly. Then, an appropriate amount of AAm, BIS, and ammonium persulfate (initiator) are dissolved into the above solvent. The pre-gel solution thus obtained is de-gassed for 20 min. The reaction is initiated by raising the temperature to 60 ${}^{\circ }C$ for 1 h. The diffusion experiments are made on a JEOL FX-60Q (JEOL, Tokyo, Japan), which is equipped with a field gradient apparatus NMPL-502 (JEOL, Tokyo, Japan), at a frequency of 60 MHz. The temperature is controlled at 30.0 ± 0.5 ${}^{\circ }C$. Details of the experimental procedure are given in the previous report [18].

**Figure 1:**
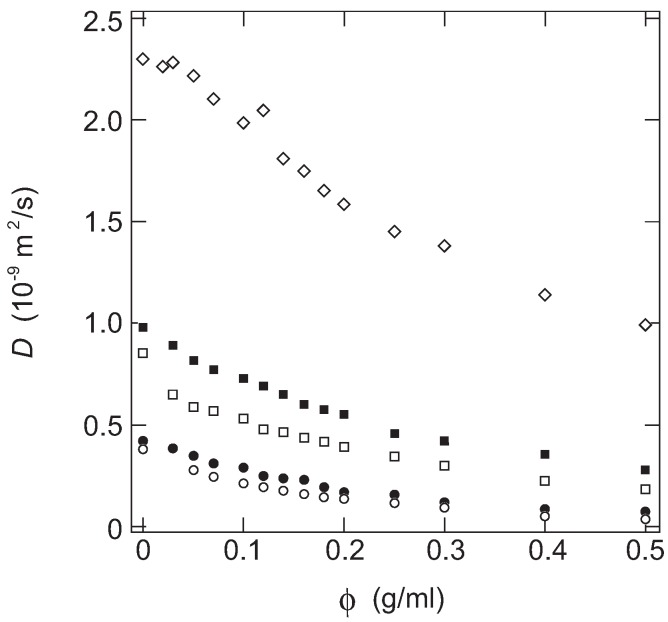
The dependence of the diffusion coefficient of probe molecules on the concentration of acrylamide (AAm) in gel. The probe molecules are water, diamonds, ethanol (filled squares), glycerin (open squares), poly(ethylene glycol) (filled circles), and sucrose (open circles), from top to bottom. The data points at the position of zero concentration represent the diffusion coefficient of each probe molecules in water that are measured by the same experimental setup. The temperature is fixed at 30.0 ± 0.5 ${}^{\circ }C$.

In Figure 1, the diffusion coefficient of the probe molecules in PAAm gels are plotted as a function of the concentration of gel.

The probe molecule, when dissolved in a simple fluid, thermally fluctuates in time and space. The thermal fluctuation and the hydrodynamic friction due to the surrounding fluid, *ζ*, determine the rate of diffusion. The diffusion coefficient, $D_{0}$, of the probe molecule of the hydrodynamic radius, $R_{h}$, in a simple fluid of viscosity, *η*, is expressed by the so-called Stokes–Einstein relationship as follows [19,20]:

(1) $$D_{0}=\frac{k_{B}T}{\zeta }=\frac{k_{B}T}{6\pi \eta R_{h}}.$$

Here, $k_{B}$ and *T* represent the Boltzmann’s constant and temperature. It is clear from the above equation that the diffusion coefficient of the probe molecule is essentially determined by the size of the probe molecule, $R_{h}$, when it is dissolved in a simple fluid at the constant temperature. However, it is found from Figure 1 that the diffusion coefficients of all probe molecules decrease monotonically with the concentration of AAm in the gel. The diffusion coefficient of the probe molecules decreases even at a very small concentration region of gel, *ca.*, less than 0.1 g/mL. The results obtained here, therefore, indicate that the diffusion of the probe molecule within the gel is far from the free diffusion in a simple fluid even in a tenuous gel. It is clear from Figure 1 that the diffusion coefficient of the probe molecule is a decreasing function of the molecular weight of the probe molecule, and it is also a decreasing function of the concentration of the gel. Furthermore, it may be clear that the concentration dependence of the diffusion coefficients of the probe molecules are similar to each other. It strongly suggests the presence of a universal function for the diffusion coefficient of the probe molecules in the gel. Since both the concentration of the gel and the size of the probe molecule is systematically changed in this study, such a function is written by these two parameters.

A similar phenomena has been also studied in semi-dilute polymer solutions and effort is devoted to finding the universal physical picture of the diffusion in these condensed polymer systems of the semi-dilute solution and gel [21,22]. Among others, one of the hopeful analyses of the diffusion processes in gel is the scaling approach [23]. In modern statistical theory of polymer systems, many physical quantities are expressed in terms of the scaling function, f(x). The diffusion coefficient of the probe molecules in gel that is normalized by $D_{0}$ is also expected to be expressed by a scaling function as follows:

(2) $$\frac{D}{D_{0}}=f\left(x\right).$$

Here, *D* and $D_{0}$ represent the diffusion coefficient of the probe molecule in the gel and that in the simple fluid, and *x* is the non-dimensional scaling variable. Since the diffusion coefficient is determined by the linear size of the probe molecule, $R_{h}$, one of the candidates for the scaling variable is a ratio of $R_{h}$ and the correlation length. The correlation length of the polymer network of gel, *ξ*, is expressed as a function of the concentration of gel, ϕ, as follows [23]:

(3) $$\xi \propto \phi^{-3/4}.$$

On the other hand, the size of the molecules with simple and compact structures is simply assumed as follows using the molecular weight of the probe molecule, *M*:

(4) $$R_{h}\propto M^{1/3}.$$

We, therefore, assume the following scaling variable:

(5) $$x=\frac{R_{h}}{\xi }\propto M^{1/3}\phi^{3/4}.$$

In other words,

(6) $$\frac{D}{D_{0}}\propto f\left(M^{1/3}\phi^{3/4}\right).$$

**Figure 2:**
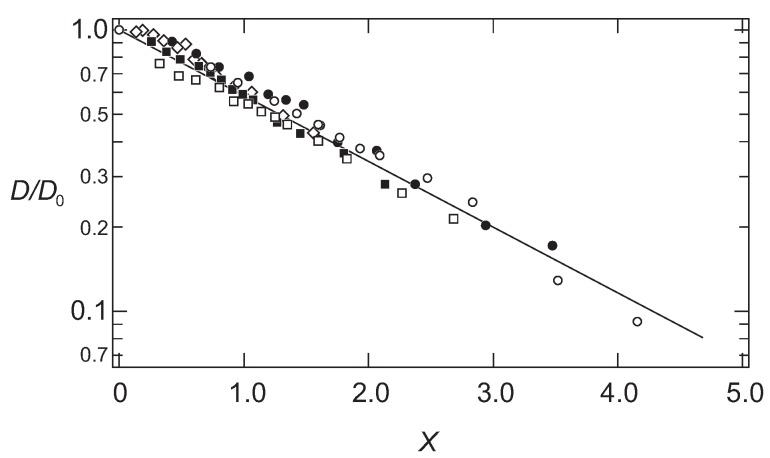
The normalized diffusion coefficient of the probe molecules in gel, $D/D_{0}$, is plotted as a function of the scaling variable, $x=M^{1/3}\phi^{3/4}$, in a semi-logarithmic manner. Symbols are the same as that of the Figure 1.

The results shown in Figure 1 are, therefore, plotted as a function of the scaling variable, *x*, in Figure 2.

It is found that all data fall onto a single master curve. In addition to this, the results are well explained by a simple functional form as follows:

(7) $$\frac{D}{D_{0}}=\exp\left(-\frac{R_{h}}{\xi }\right).$$

The results are in good agreement with the hydrodynamic calculations for the scaling function [21,22]. It is found that the diffusion coefficient of the probe molecules follows the simple scaling theory.

**Figure 3:**
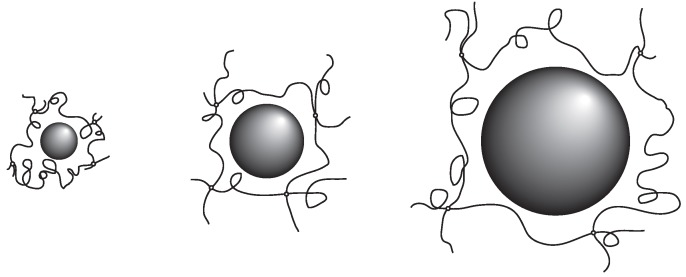
The schematic representation of Equation (7). The size of the probe molecules is different, but the ratio of the size and the correlation length, $R_{h}/\xi$, is similar in these three cases. The diffusion coefficient of the probe molecule that is normalized by the diffusion coefficient of the probe molecule in water, $D/D_{0}$, is almost the same in these cases.

The results obtained here suggest strongly that the probe molecule that diffuses in the polymer network of PAAm gel mainly experiences resistance due to the polymer network. In addition, the effects of the polymer network on the diffusion of the probe molecules are determined by the ratio of the correlation length of the polymer network and the radius of the probe molecule. The results in Equation (7) indicate that the relative rate of the diffusion is equivalent if the ratio of the hydrodynamic radius and the correlation length of the polymer network, $R_{h}/\xi$, are equal. The smaller probe molecule diffuses faster within the gel. However, even the size of the probe molecule is larger, it diffuses in the gel at the similar rate as the smaller probe molecules if the mesh size of the gel is large enough. The rate of the diffusion in the gel is, in the simplest case, determined by the geometrical factor of the probe molecule and the polymer network of the gel. The parameter that determines the diffusion of the substance in gel is the ratio of the size of the probe molecule and the correlation length of gel, $R_{h}/\xi$. The results obtained here are schematically illustrated in Figure 3.

## 4. Friction of Gel

Many efforts have been devoted to creating active systems such as artificial muscles after finding the volume phase transition of gel [24]. When gel collapses, a huge amount of gel fluid is squeezed out of gel. Hence, the acceleration of the drainage of gel fluid from gel is the key to success. In other words, an understanding of the friction between the polymer network of gel and gel fluid is required. The study of the kinetics of the swelling of gel indicates that the swelling of gel is determined by the collective diffusion coefficient of gel, $D_{c}$ [25,26,27]:

(8) $$D_{c}=\frac{E}{f}.$$

Here, *E* represents the longitudinal elastic modulus of the polymer network alone and *f* the friction coefficient between the polymer network of gel and gel fluid. Equation (8) indicates that the polymer network of gel moves under the drag force of the surrounding fluid.

**Figure 4:**
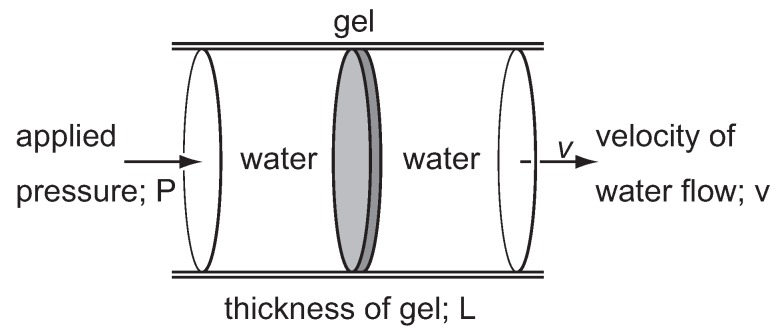
The schematic illustration of the principle of friction measurement. The gel of the thickness *L* is set in a column with the limb fixed. Then, small pressure, *P*, is applied to water, which covers the left side of the gel. The velocity, *v*, of water flowing out of gel is measured in the stationary state. The friction coefficient of gel is defined as f=P/vL.

The measurements of the friction between the polymer network of gel and gel fluid is, in principle, simple as schematically shown in Figure 4. However, only a little is known about the frictional properties of gels. The reason is simple—the friction coefficient of the polymer network is huge, while gel is fragile. Detailed study on the frictional properties of the PAAm gels are reported where the strategies to design the apparatus are also reported [28].

### 4.1. Friction of Uniform Gel

The first study we should make is to obtain all the aspects of the frictional properties of uniform gel. For this purpose, we use PAAm gel because of its many advantages as described in the previous sections.

**Figure 5:**
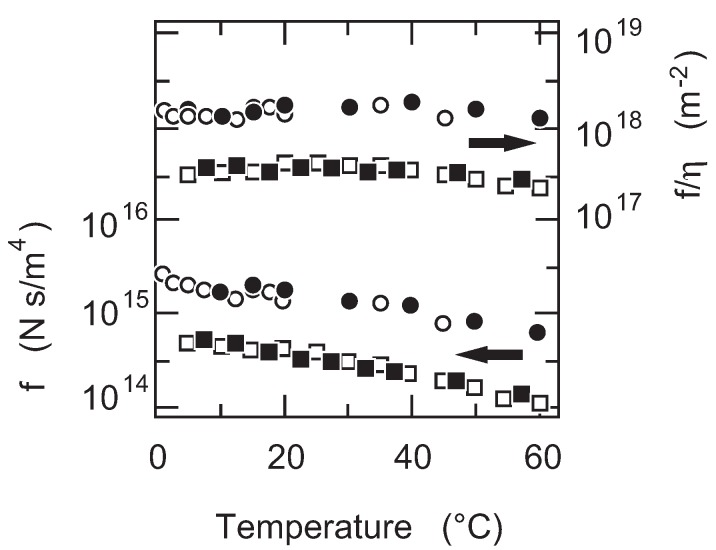
The temperature dependence of the friction coefficient of poly(acrylamide) (PAAm) gel. The concentration of gel is 5 % (squares) and 8% (circles). The friction coefficient that is normalized by the viscosity of water, f(T)/η(T) is also shown in this figure. The solid symbols are used in the increasing of the temperature, and the open symbols are used in the decreasing of the temperature.

First of all, the temperature dependence of the friction coefficient of PAAm gel is studied. The results are given in Figure 5. The friction coefficient of PAAm gel decreases monotonously with the increase in temperature as shown in Figure 5. It may be natural to assume that the friction coefficient itself depends on the viscosity of the flowing fluid. Thus, the friction coefficient of PAAm gel is normalized by the viscosity of water and the results are also plotted in Figure 5. It is found that the friction coefficient of PAAm gel is independent of the temperature when the friction coefficient is normalized by the viscosity of water, f(T)/η(T). The results obtained here indicate that the structure of the polymer network of PAAm gel is independent of the temperature. The results further indicate that the raw value of the friction coefficient includes the information on the viscosity of the flowing fluid. In the present case, the friction coefficient of PAAm gel is proportional to the viscosity of water, $f\propto \eta_{\mathrm{water}}$.

**Figure 6:**
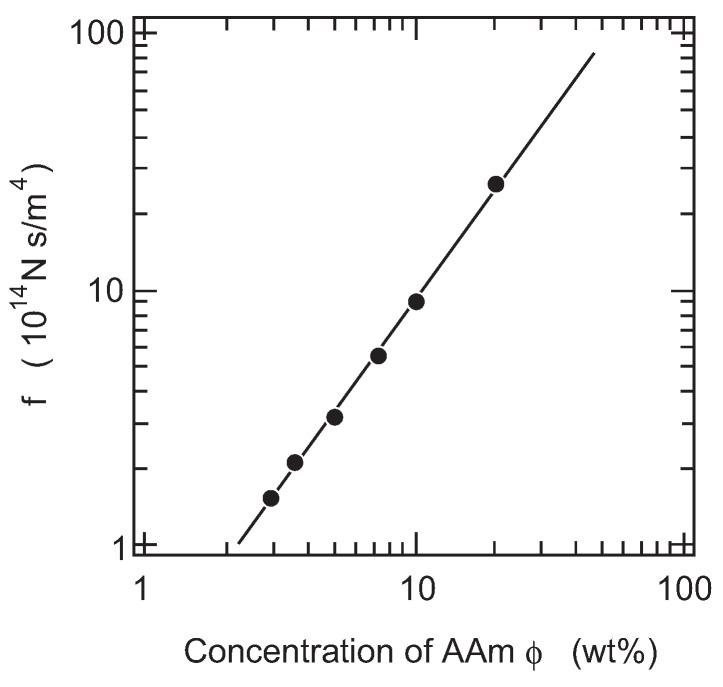
The concentration dependence of the friction coefficient of PAAm gel. The raw value of the friction coefficient is plotted as a function of the concentration of PAAm gel in a double logarithmic manner. The temperature is fixed at 20.0 ${}^{\circ }$ C in these measurements. The straight line with the slope of 1.5 is the results of the least squares analysis.

Secondly, we study the concentration dependence of the friction coefficient of PAAm gel. The raw value of the friction coefficient of PAAm gel is plotted as a function of the concentration of gel in a double logarithmic manner in Figure 6, since the measurements are made at a constant temperature of 20 ${}^{\circ }$C, and hence, the viscosity of water does not show any singular behavior at this temperature.

The least squares analysis shows that the concentration dependence of the friction coefficient is well expressed by a power law relationship as,

(9) $$f\propto \phi^{1.5}.$$

The scaling suggests that both the collective diffusion coefficient of gel and the elastic modulus of gel can be written as a function of the concentration as follows [23]:

(10) $$\begin{array}{cc} D_{c} & \propto \phi^{3/4}, \end{array}$$

(11) $$\begin{array}{cc} E & \propto \phi^{9/4}. \end{array}$$

Equation (8) with the above scaling relationships reveals that the friction coefficient of gel scales with the exponent of 6/4 for the power law relationship. The concentration dependence of the friction coefficient obtained here is in good agreement with the scaling theory. The results are also explained from the molecular structure of gel. When the pressure is applied to the system, water flows through the open space of the polymer network of gel. The gel is, therefore, regarded as the assembly of the pore. The rate of water flow is assumed to be proportional to the viscosity of the fluid, *η*, and is inversely proportional to the cross section of the pores. The cross section of the pores in gel is reasonably assumed to be proportional to the square of the correlation length of gel, *ξ*:

(12) $$f\propto \frac{\eta }{\xi^{2}}.$$

It is suggested by the scaling theory that the correlation length of the polymer network depends on the concentration of gel as follows [23]:

(13) $$\xi \propto \phi^{-3/4}.$$

The scaling again results as $f\propto \phi^{1.5}$. Equation (12) is rather useful when the friction coefficient of gel is discussed in terms of the structure of the gel.

**Figure 7:**
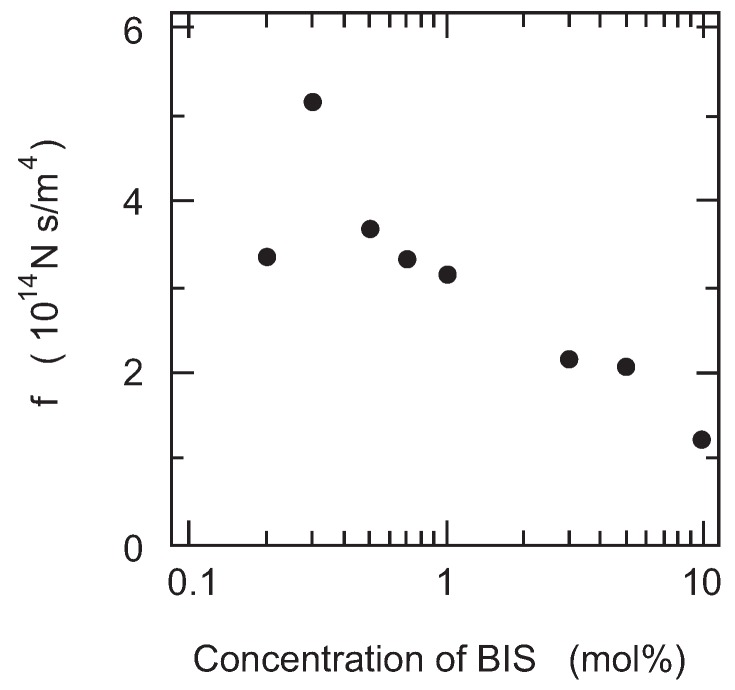
The cross-linker concentration dependence of the friction coefficient of PAAm gel. The total concentration of gel is fixed at 700 mM, and the mole fraction of the cross-linker is varied.

Finally, the friction of PAAm gel is measured as a function of the cross-linking density. It is expected that the size of the pores in the polymer network of gel decreases with the cross-linking density of gel. In other words, the correlation length of the polymer network of gel decreases with the concentration of BIS. Therefore, it may be natural to assume that the friction coefficient of gel increases with the concentration of BIS according to Equation (12). However, the results, as shown in the Figure 7, clearly indicate that the friction of PAAm gel decreases with the concentration of BIS. In addition, it is also found that gel becomes translucent with the concentration of BIS and finally the gel becomes completely opaque when the concentration of BIS exceeds 3 % in mole fraction.

The increase in turbidity of the gel indicates the appearance of the density fluctuations in the polymer network of gel. The density fluctuations that appear in the present system are irreversible and independent of time, and hence, it indicates that the polymer network of gel itself is spatially non-uniform. In contrast, gel is transparent as long as the mole fraction of BIS is less than 3%. The friction of gel, however, clearly decreases with the concentration of BIS even in the concentration region where the transparent gels are obtained. These results thus strongly suggest that the seeds of the density fluctuations emerge in the polymer network of gel at a lower concentration region where gel looks completely transparent. The frictional property of gel, therefore, reflects the spatial homogeneity of gel in an extremely sensitive manner. However, we have to wait for the advancement of new technology that provides us with observations of the structure of the polymer network of the opaque gels in real space, such as the confocal laser scanning microscope (CLSM), for further detailed discussion of the phenomenon. We will return to this point again in the later section.

### 4.2. Friction of Non-Uniform Gel

**Figure 8:**
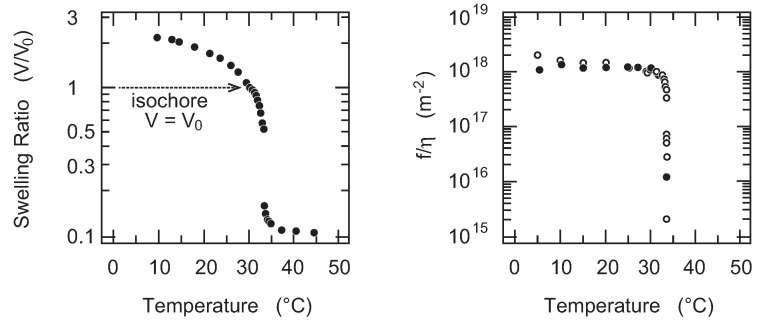
The temperature dependence of the swelling ratio of gel (left; $V/V_{0}$) and that of the friction coefficient of poly(*N*-siopropylacrylamide) (PNIPA) gel that is normalized by the viscosity of water (right; f(T)/η(T)). The friction is measured under a constant volume condition that is shown in the left figure as an arrow. Open circles are used in the increasing of the temperature and solid circles are used in the lowering of the temperature.

Many studies have been devoted to clarifying the relationship between the chemical structure of the monomer unit and the biological phenomena [29,30]. Among others, the finding of the volume phase transition in poly(*N*-isopropylacrylamide) gel (PNIPA gel) is of importance since PNIPA gel shows the volume phase transition in response to the temperature change under water [31]. The transition in PNIPA gel is thought to be a result of the hydrophobic interaction between the *N*- isopropylacrylamide (NIPA) residues in the polymer chain. According to the fact that PNIPA gel shows the volume phase transition in water, the gel is widely used to study the aspects of the volume phase transition of the gel, for instance, such as the critical kinetics of the volume phase transition of gel [32]. One of the unsolved phenomena currently is the critical behavior of the friction of gel in the vicinity of the volume phase transition point. It is expected in the early stage of the studies of the volume phase transition of gel that the friction of gel becomes smaller in the vicinity of the volume phase transition point of gel [9]. Therefore, the critical behavior of the friction is studied in the vicinity of the volume phase transition temperature of PNIPA gel [33]. The results are given in the Figure 8. The results clearly indicate the drastic decrease of the friction coefficient of gel in the vicinity of the volume phase transition temperature. The friction coefficient of gel decreases more than three orders of magnitude as the temperature approaches the volume phase transition temperature. The change of the friction coefficient of gel is reversible against the temperature change. The results indicate that the reversible decrease of the friction coefficient of gel is caused by the emergence of the critical density fluctuations in the polymer network of gel. The characteristic time scale for the density fluctuations becomes longer in the vicinity of the phase transition point, and hence, the polymer network of gel looks like an assembly of the large pore of the radius that corresponds to the correlation length of the density fluctuations, *ξ*. Water flows through the open space that is created by the lower density regions of the polymer network, avoiding the denser region that blocks the flow. It is reasonably assumed that the correlation length of the density fluctuations diverge in the vicinity of the volume phase transition point. The friction of the polymer network of gel, therefore, becomes smaller and it practically vanishes at the phase transition point. The critical exponent for the divergence of the correlation length near the critical point is also estimated from these results. We, however, observed a relatively large discrepancy with the expected value. It is clear from the swelling curve of gel that is shown in Figure 8 that the isochore conditions employed here do not hit the exact critical point of gel. Therefore, the critical exponent we obtained here corresponds to the off-critical value. Although the critical slowing down is observed by the friction experiment of PNIPA gel, the critical behaviors of gel is still open to question.

### 4.3. Friction of Colloid Gel

It is found that the friction of PAAm gel decreases with the cross-linking density in the previous section. The results suggest strongly that the non-uniform density fluctuations are frozen into the polymer network of gel whenever the cross-link is introduced. The structure of the polymer network of gel, therefore, may change if the concentration of BIS is increased further. The detailed study on the structure of the polymer network of gel, therefore, should be made firstly. Then, the relationship between the structure and the frictional properties of gel is discussed.

The formation of PAAm gel has been studied in detail by changing the concentration of AAm and BIS drastically. The results are expressed as a kind of phase diagram [34]. It has been found that PAAm gel becomes turbid at higher concentration regions of BIS. Therefore, the turbidity change of PAAm gel itself is a well known phenomenon. The structural study of opaque gels at these concentration regions has been also made by using spectroscopy techniques [35]. Although these studies strongly suggest the formation of the cluster of BIS in the opaque gel, the structure of the opaque PAAm gel in real space could not be obtained.

**Figure 9:**
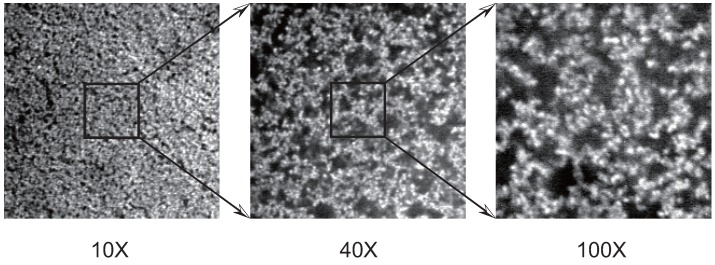
The confocal laser scanning microscope (CLSM) images of opaque PAAm gel. The magnification of the image is 10×, 40×, and 100× from left to right. The length of the side of these images is 189 m, 48.8 m, and 18.9 m from left to right. The concentrations of AAm and *N,N*-methylenebisacrylamide (BIS) are 350 mM. The total concentration is 700 mM and the mole fraction of BIS is 50%. The squares in the image of 10× is the observation area of 40× observation. The square in the image of 40× is the observation are of 100× observation. These squares only show the relative size of the images and are shown only for a guide to the eye.

The idea of the confocal scanning microscopy has been reported in 50’s [36]. It became a popular technique when the laser was used as the light source in the 1980s as the confocal laser scanning microscope (CLSM). Firstly, CLSM was used in the research area of biology, and eventually, it spread to the research area of materials science. In the area of gel science, the structure of PNIPA gel was firstly studied using CLSM. It is well known that PNIPA gel also becomes opaque if the reaction temperature is raised to 30 ${}^{\circ }$C or more. The network structure of such opaque PNIPA gels is solved by CLSM imaging technique [37]. We also study the structure of the opaque PAAm gel [38]. In Figure 9, CLSM images of the opaque PAAm gel are shown.

**Figure 10:**
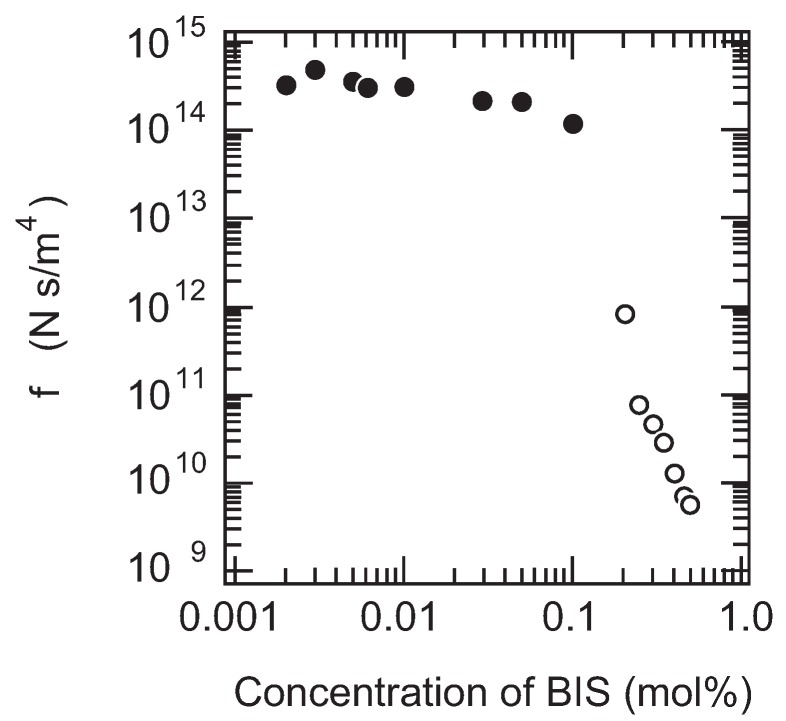
The cross-linker concentration dependence of the friction coefficient of PAAm gel at higher concentration region of BIS (open circles). The results shown in Figure 7 are also plotted in this figure (solid circles). The gels are prepared at a total concentration of 700 mM, and the mole fraction of the cross-linker is changed.

The total concentration of PAAm gel observed here is 700 mM and the mole fraction of the BIS is 50%. The images of the same gel are gained at various magnifications. The magnifications of the images are 10×, 40×, and 100×. The brighter portion in the images corresponds to the dense region of AAm and BIS, and the darker region is the dilute region of AAm and BIS. It is clearly shown that the opaque PAAm gel consists of the aggregate of the colloidal particles from these images. The detailed analysis of the confocal images of the opaque PAAm gel yields that the density of the particle is of the order of $1\times 10^{3}$ kg/m${}^{3}$. The density of the particle is almost the same as that of AAm and BIS in the solid state. Both AAm and BIS are densely polymerized into a sphere of the colloidal dimension. The particles thus formed in the opaque gels are, therefore, regarded as a hard sphere. In addition to this, the small angle neutron scattering from the opaque gel strongly suggest that gel is a fractal with the fractal dimension of $D_{f}≃2.7$ [39,40,41,42]. The results suggest that the colloidal particles of AAm and BIS are formed and they aggregate by the process of the diffusion limited aggregation or the cluster-cluster aggregation process. We expect that water can easily flow through the darker portion in the colloidal gel since the mass is mostly localized in the colloidal particles, and, hence, the darker portion of the image serves as the open pore for the fluid flow. The friction experiments are made in the higher concentration region of BIS in gel. The results are given in Figure 10 [43].

It is found that the friction coefficient of gel decreases more than four orders of magnitude when the mole fraction of BIS is increased to 0.2 or more. The opacity of gel increases at the same concentration of BIS where the drastic decrease of the friction occurs. This suggests strongly that the structure of the polymer network of gel changes from the uniform polymer network to the colloidal aggregate. In accordance with the structural transition of the polymer network of gel, the friction of gel decreases more than four orders of magnitude. The friction of the opaque gel can be explained in terms of the structure of gel because the structural parameters can be deduced from the CLSM images. First of all, the density of the particle is high, and is almost the same as AAm and BIS in a solid state. Secondly, the particle is more or less spherical in shape. The particles are regarded as hard spheres. Water thus flows around the aggregates of hard spheres experiencing the hydrodynamic friction. Since the density of the sphere is almost the same as that of the solid, the aggregates only behave as the fixed obstacles for water flow. If only one solid particle of radius *R* is suspended in the flowing fluid, the hydrodynamic friction due to the particle is expected to be $f_{\mathrm{obstacle}}=6\pi \eta R$. In the colloid gel, however, *N* particles are included in a unit volume of gel. The friction of the colloid gel thus becomes

(14) $$f_{\mathrm{obstacle}}=N6\pi \eta R.$$

Here, *N* represents the number of colloid particle in a unit volume of gel. Both *N* and *R* are calculated from the CLSM images of gel that are used as the samples of the friction experiments. Then, the friction of gel is calculated by using Equation (14). The calculated values show the same concentration dependence with the experimental results. The numerical values are, however, about one-half of the experimental values. One of the reasons for this discrepancy arises from the resolution limit of the CLSM that the focal plane of the CLSM has a finite thickness. In the case of the present system, the thickness of the focal plane is about 1 m according to the manufacturer. In addition, the size of the particle in the present case is comparable with the thickness of the focal plane. Therefore, the overlap of the particles in the depth direction in the CLSM image is unavoidable. The number of the particle, *N*, that is simply calculated from the CLSM image therefore underestimates the actual number of the particle contained in the image. The friction of gel thus calculated becomes smaller than the actual values of the friction. The details are given in the previous reports [43,44].

## 5. Conclusions

We review the probe diffusion of gel and the hydrodynamic friction of the polymer network. It is found that the diffusion coefficient of compact molecules in gel is well described by the scaling theory. The essential parameter that determines the diffusion coefficient is the ratio of the tow sizes, R/ξ, where *R* is the radius of the probe molecule and *ξ* the mesh size of the polymer network. In the study, most probe molecules are rather small and compact, and, hence, the scaling results are natural. In addition to this, it is also interesting that the diffusion coefficient of poly(ethylene glycol) follows the same scaling law since poly(ethylene glycol) is a linear polymer. In designing the diffusion experiments, we expected different diffusion mechanisms for poly(ethylene glycol), that is, the diffusion by the reptation [45,46]. The molecular weight of poly(ethylene glycol) used in the diffusion experiments is 200, which indicates that the polymer consists of only about five monomers. The results indicate that polymers of this size can be regarded as compact molecules. Hence, the diffusion experiments using the longer linear polymers is of interest for establishing the entire physical picture of the diffusion in gel. Since we have obtained the simplest view of the diffusion in gel, it may be of interest to study the system where the probe molecule interacts with the polymer network strongly through the typical interactions such as the hydrophobic interaction, the electrostatic interaction and so forth. Further detailed study on the diffusion in gel is required.

The results of the frictional properties of the polymer network of gel is also easily explainable by the scaling theory. The friction of the polymer network is essentially determined by the mesh size of gel. The measure of the mesh size of gel is the correlation length of the polymer network, *ξ*. In Equation (12), however, we only assume the proportionality between the friction coefficient and the correlation length, $f\propto \eta /\xi^{2}$. Thus, the numerical coefficient of this equation is not clear at present. It is well known that the Hagen–Poiseuille flow is one of well defined flow in hydrodynamics:

$$P=8\eta L\frac{v}{r^{2}},$$

where *P*, *L*, *r*, and *v* are the hydrodynamic pressure, the length of the capillary, the radius of the capillary, and the velocity of the flowing fluid. Taking into account Equation (12), one obtains the proportional constant of eight for the friction of the capillary of radius *r* [47]. It seems that the proportional constant for Equation (12) depends on the structure of the system. Therefore, it will contribute in designing the flow through the porous medium if the numerical coefficient of Equation (12) is clarified. The frictional studies of the well defined gel or the combined experiments of the structure and the friction as well as the theoretical studies are required for understanding the flow processes in gel.

The diffusion of the molecule, which is dissolved in gel fluid, and the flow of gel fluid are independent phenomena from each other because the origin of the driving force for each process is not the same. Therefore, both transport processes can occur simultaneously in a system as is seen in the pattern formation in the shrinking gel. The well defined experimental study of such systems is of interest for obtaining information on the general transport phenomena in the soft material. For instance, it is expected that the crossover from the transport by flow to that by diffusion will occur at an extremely low pressure gradient. The detailed study of the crossover phenomena may open new insights into transport phenomena in biological systems.

## Abbreviations

The following abbreviations are used in this manuscript:

PFG-NMR: Pulsed Field Gradient Nuclear Magnetic Resonance
AAm: Acrylamide
PAAm gel: Poly(acrylamide) gel
NIPA: *N*-isopropylacrylamide
PNIPA gel: Poly(*N*-isopropylacrylamide) gel
BIS: *N*, *N*-methylenebisacrylamide (cross-linker)
CLSM: Confocal laser scanning microscope
